# Data mining application to healthcare fraud detection: a two-step unsupervised clustering method for outlier detection with administrative databases

**DOI:** 10.1186/s12911-020-01143-9

**Published:** 2020-07-14

**Authors:** Michela Carlotta Massi, Francesca Ieva, Emanuele Lettieri

**Affiliations:** 1 MOX Laboratory, Department of Mathematics, Politecnico di Milano, Via Bonardi 9, Milan, Italy; 2 CADS - Center for Analysis, Decisions and Society, Human Technopole, Palazzo Italia, Via Cristina Belgioioso 28, Milan, 20157 Italy; 3 CHRP-National Center for Healthcare Research and Pharmacoepidemiology, Università degli Studi di Milano-Bicocca, via Bicocca degli Arcimboldi 8, Milan, 20126 Italy; 4 Department of Management Engineering, Politecnico di Milano, Via Lambruschini 4/c, Milan, 20100 Italy

**Keywords:** Data mining, Fraud, Upcoding, Administrative database, DRG

## Abstract

**Background:**

The healthcare sector is an interesting target for fraudsters. The availability of a great amount of data makes it possible to tackle this issue with the adoption of data mining techniques, making the auditing process more efficient and effective. This research has the objective of developing a novel data mining model devoted to fraud detection among hospitals using Hospital Discharge Charts (HDC) in Administrative Databases. In particular, it is focused on the DRG upcoding practice, i.e., the tendency of registering codes for provided services and inpatients health status so to make the hospitalization fall within a more remunerative DRG class.

**Methods:**

We propose a two-step algorithm: the first step entails kmeans clustering of providers to identify *locally consistent* and *locally similar* groups of hospitals, according to their characteristics and *behavior* treating a specific disease, in order to spot outliers within this groups of peers. An initial grid search for the best number of features to be selected (through Principal Feature Analysis) and the best number of local groups makes the algorithm extremely flexible. In the second step, we propose a human-decision support system that helps auditors cross-validating the identified outliers, analyzing them w.r.t. fraud-related variables, and the complexity of patients’ casemix they treated. The proposed algorithm was tested on a database relative to HDC collected by Regione Lombardia (Italy) in a time period of three years (2013-2015), focusing on the treatment of Heart Failure.

**Results:**

The model identified 6 clusters of hospitals and 10 outliers among the 183 units. Out of those providers, we report the in depth the application of Step Two on three Hospitals (two private and one public). Cross-validating with the patients’ population and the hospitals’ characteristics, the public hospital seemed justified in its outlierness, while the two private providers were deemed interesting for a further investigation by auditors.

**Conclusions:**

The proposed model is promising in identifying anomalous DRG coding behavior and it is easily transferrable to all diseases and contexts of interest. Our proposal contributes to the limited literature regarding *behavioral models* for fraud detection, identifying the most ’cautious’ fraudsters. The results of the first and the second Steps together represent a valuable set of information for auditors in their preliminary investigation.

## Background

Being Healthcare the target of large public and private investments, this sector is appetible for frauds. A 2017 OECD report lists some of the major worldwide frauds related to Healthcare [1]. Among fraudulent behaviors, the upcoding practice has a paramount economic impact on Healthcare [2–5]. It consists in the classification of a patient within a Diagnosis Related Group (DRG), that produces higher reimbursements [6]. When a patient is hospitalized by a provider, such provider registers all diagnosis and interventions within the Hospital Discharge Chart (HDC), that affects the final DRG of the single hospitalization once elaborated by the *grouper*^1^. The provider may therefore have the incentive to alter the registrations, to make the hospitalization fall into a more remunerative DRG. While this practice is more likely due to unintentional errors by coders in public hospitals, in private hospitals or private medical practices it might be actually due to profit maximization purposes [7, 8]. Given the ever-growing availability of digital data, the adoption of data mining techniques might support the manual audit process for fraud detection in an efficient and effective way.

A small number of studies tried to tackle fraud detection by adopting supervised techniques [9–12], which despite their undeniable potential and predictive power, exhibit the risk of focusing on old patterns and losing predictive capability as new records are evaluated over time [13]. Due to these considerations, most of the available literature in the field focuses on unsupervised techniques [3, 5, 14–23], with the limitation of spotting providers with very high claiming episodes, which distinguish themselves as *evident* outliers [23]. However, types of fraud are growing increasingly sophisticated. Patterns detected from fraudulent and nonfraudulent behaviors become rapidly obsolete, while fraudulent providers are becoming smarter in finding more cautious approaches to prevent investigation [19], as they do not appear within the *evident outliers*’ groups [24]. The unsupervised behavioral models suggested in [17] and [19] try indeed to respond to this limitation. As mentioned in [17], There are two major reasons why fraud investigation within unsupervised databases does not usually involve these type of models. First, even though there is no formal comparison, it is likely that behavioral models flag more false positives than methods based on searching for *extreme* outliers, and this increases costs to the healthcare system. Secondly, the monetary amounts recovered by the agencies might be smaller when dealing with *cautious* fraudsters. However, the utilization of both types of model together would provide great value to the healthcare system, and literature should devote the proper attention to the cost of false negatives and the dynamic nature of fraud.

**Fig. 1:**
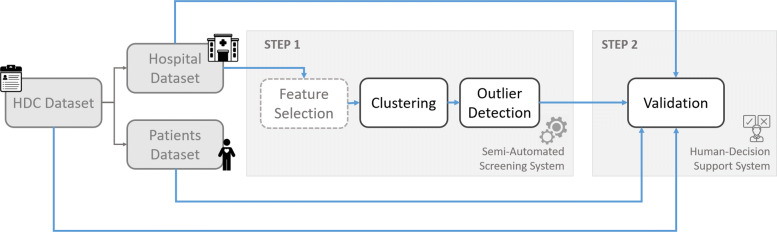
Process Flow. Schematic representation of the algorithm’s process flow. The first three grey boxes on the left represent the data sources. From HDC Dataset we derived the two additional datasets (Patients and Hospitals). Only Hospital Dataset enters Step 1, while all three of them are retrieved for Step 2

This study indeed lies in the small group of methods tailored to support the identification of those less *evident* fraudulent providers, while taking into consideration the risk of false positives. Specifically, it aims at proposing a novel methodology to support auditors in the preliminary phases of screening providers to spot suspects eligible for investigation. We adopted an unsupervised *behavioral* approach, to spot even the most cautious fraudsters, and built a robust, scalable and user-friendly algorithm. The overall method (Fig. 1) entails both a semi-automated funneling step that identifies a list of potential fraudsters, followed by a human-decision support system that specifically aim at helping auditors in discriminating between actual and false positives. Because of the larger and larger use of Administrative Databases by auditors, we decided to leverage on these data as source of information. Our algorithm is coded using Python and the code is available by the authors upon request.

## Methods

The idea behind our methodology is that *cautious* fraudsters cannot be found searching for outliers among the entire population of providers together. Providers indeed differ significantly among one another, due to size, technological endowment, resources, specialization etc. However, fraudsters might be hard to spot among groups similar on those dimensions only. Therefore, we are interested in isolating suspects among groups of hospitals expected to behave similarly (*Step One*). Then, we add an additional step (*Step Two*), that has the objective of helping auditors in the validation of results, by checking some fraud-related measures, and the complexity of the patients treated by the outliers, understanding whether their *outlyingness* might be justifiable in that sense. This reduces the risk of false positives being investigated even deeper.

### Data

Our model was developed and tuned on Administrative Data (originated from HDC) about hospitalizations for Heart Failure (HF) happened in the Lombardy Region within the timespan 2013-2015. The extraction procedure is reported in the pseudo-code in Algorithm 1 in Table 1. Data were already anonymized and both patients’ and hospitals’ confidentiality were preserved. Note that the methodology here applied to HF can be easily transferred to any other disease.

**Table 1:** Pseudo-code for the HDCs extraction process

	

### Datasets Preprocessing

Data contain 396,246 events (hospitalizations) related to 132,254 patients, experiencing at least one HF episode in the timespan 2013-2015. Once extracted, the information had to be reshaped and aggregated in the following datasets.

*HDC Dataset*. This dataset has one row per event (hospitalization, multiple events per patient are allowed), reporting information about the patient and the treatments or diagnosis he/she encountered. Each record was subsequently associated with the Comorbidity Index (CI) of the patient for that hospitalization, adopting the Combined Comorbidity Score in [25].

*Patients Dataset*. This aggregates all available information within the HDC Dataset at patient level. Each row represents one patients who experienced an HF episode in the timespan of interest. The aforementioned CIs per HDC were here summed together to estimate the general health status of the subject [25]. For those variables where more than a value was available (e.g. each HDC reports the ’age’ information), we decided to summarize the information into a single value (e.g. the age at first hospitalization). In Appendix A.1 the whole list of variables in the dataset is provided [see Additional file 1].

***Hospitals Dataset***. This reports information with the single hospital as statistical unit. The dataset contained 183 facilities visited by at least one patient. It required a procedure aimed at including relevant information to identify a fraudulent behavior by the providers. Using the variables found within literature as a reference, all information available within the HDC Dataset was aggregated for each hospital (e.g. cost per hospitalization [4, 15–17, 23], average Length Of Stay (LOS) [26], number of episodes in a given period of time [23], etc.). Then, a set of additional indexes were estimated. The *behavioral* aspect of our representation of each provider was inspired by the work of Ekin et al. [27]. On the basis of how often each HF-DRG (*i*) was registered by each provider (*h*), we estimated a set of variables for each provider (**r**_*ih*_), that could represent a good proxy of how hospitals behaved in the treatment of HF. Details about the variables of the Hospital Dataset are provided in Appendix A.2 [see Additional file 1].

### Model Proposal

Given the outcome of the data preprocessing, we are now able to propose a two-step algorithm for screening hospital behavior and detecting potential *upcoding* fraud.

**Step One.** The first step aims at grouping providers on the basis of how they behaved in the treatment of HF-affected patients, and isolating potential fraudsters that separate themselves from the performances of their reference peers. Note that, in a totally unsupervised setting, it is not possible to make any prior assumption about whether any actual clusters of providers exists within the available observations. In the attempt to spot *cautious* fraudsters that might be hidden within the whole group of hospitals, we seek for providers that behave differently from the peers they are supposed to be aligned with. This translates into seeking for the most extreme points (i.e. *outliers*) within *locally consistent* groups of observations. In other words, in looking for points that are possibly closer to the points belonging to their group, w.r.t. how close they are to other groups. This concept is based on the same assumption of the *Silhouette Score* of the K-means algorithm [28]. For this reason, this step entails the application of a K-means on the Hospitals Dataset. K-means indeed allows us to define these locally consistent subgroups by automatically subsetting data via Euclidean Distance into K hypershperical groups with potentially different size and density, while trying to maximize the *Silhouette* of the identified local groups.

Since this algorithm may be applied to contexts where the number of observations (hospitals) could be similar to or smaller than the number of variables to be used for clustering, we include in this step a robust feature selection method. Considering the unsupervised setting, for this task we chose an unsupervised feature selection method, specifically the Principal Feature Analysis (PFA) proposed in [29] and run it *B*=200 times. At each run the algorithm selects a subset of *n* most relevant features. Because of the greedy nature of this procedure, we counted how many times each variable appeared in 200PFA iterations, and we kept as the final selected variables the top *n* most appearing features. The clustering of hospitals’ data in our model will therefore be influenced by the choice of the number of features to be selected (*n*), and the number of clusters (*k*) imposed to the k-means algorithm. We addressed this matter by including a grid search of the best couple of parameters. Our algorithm iterates over features selection and clustering for all possible combinations of *k* and *n*, among a list of reasonable values, and selects the two parameters that maximize the average silhouette width of the resulting clusters. It has to be mentioned that the feature selection passage is not mandatory, in case the number of observations is sufficiently higher than the number of features, and/or the user is not familiar with such kind of procedure. This first part of Step 1 is described in Algorithm 2 in Table 2.

**Table 2:** Pseudo-code for the first part of Step 1 Algorithm

	

Once defined the best model and performed the clustering, the algorithm identifies the outlying providers w.r.t. their similar peers, i.e. the clusters they belong to. Specifically, the Euclidean distance between each hospital and the centroid of its corresponding cluster is computed, and the hospitals with a distance above a specified threshold are selected and flagged as outliers. Here we chose the 95th percentile of the distances’ distribution, a trade-off choice to keep the number of outliers manageable by controllers. This second passage of Step 1 is described in Algorithm 3 in Table 3.

**Table 3:** Pseudo-code for the second part of Step 1 Algorithm

	

**Step Two.** The second step focuses on the deeper investigation of suspects labeled as outliers. Since behavioral methods demonstrate to have high false positive rates [17] - even though stronger in identifying cautious fraudsters - a further validation of results is recommended. This validation step aims at supporting auditors in veryfing whether the outlyingness of the aforementioned hospitals (mainly based on features which are proxies of hospitals behavior, like the *r*_*hi*_ values), might be also explained by specific features useful to evaluate a fraudulent behavior in the healthcare domain.

Specifically, we recommend to use 4 variables to verify their suspiciousness w.r.t. *upcoding* fraud: degree of specialization (HF-related hospitalizations among all HDCs, proxy of attractiveness for a HF patients), percentage of DRGs with complications (CC), Upcoding Index (Appendix A.2, Additional file 1 - Formula F.A.1), and average cost per patient. In addition, we decided to include in the analysis the Number of Visits each provider received, as it helped in giving better interpretations to the other fraud-related measures. All these variables can be found within the Hospital Dataset, as detailed in Additional file 1: Appendix A.2. We propose here a visual dashboard displaying the distribution of each dimension for the whole population of providers, highlighting the outliers’ position w.r.t. the others in terms of percentiles.

A further validation of the suspects identified in Step One derives from analyzing the patients’ population treated by the hospital, using the Patients Dataset and the HDC Dataset. The idea behind is to verify whether the anomalous behavior may be justified by the different complexity of the treated population. To perform this passage we use the variables in Table 4. We built another set of visualizations to provide auditors with an easy and understandable tool to support their decisions. In this case, plotting the distribution of the variables in Table 4 for the outliers under scrutiny and the entire population, allows for the comparison of one specific hospital’s patients’ case mix and the costs it faced, w.r.t. the others.

**Table 4:** Variables of interest for cross validation

**Variable**	**Dataset**	**Notes**
Age	Patients	Indicator of patients’ complexity
length of Stay	Patients, HDC	Indicator of patients’ complexity
Comorbidity	Patients, HDC	Indicator of patients’ complexity
Total Costs	Patients, HDC	Patients’ expensiveness
Cost / length of Stay	HDC	Expenses in relation with intensity of care
Cost / Comorbidity	Hospital	Expenses in relation with intensity of care

Step 2 can be considered as a visual support tool provided to human decision-makers (i.e. auditors), in the phase of scrutinizing the results of the previous phase. The reshaped information it provides serves as a powerful tool to help them actively mitigating the risk of investigating deeper some potential false positives arising from the previous automated step.

In Fig. 1 we represented a schematic version of the whole process flow, including data preprocessing, followed by the two steps.

## Results

**Fig. 2:**
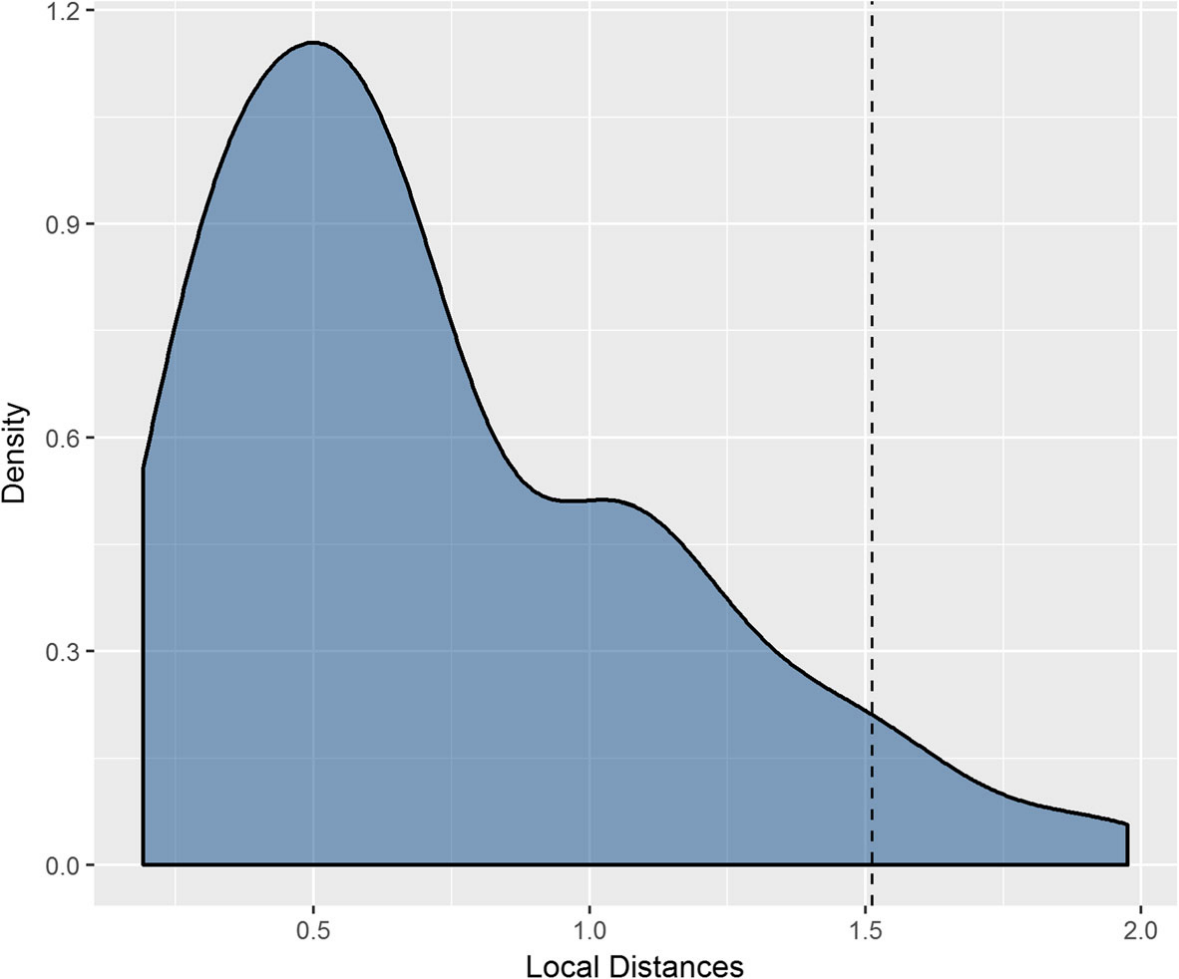
Distribution of Local Distances and Threshold. Local distances distribution of hospitals from the center of their cluster. The 95th percentile threshold is highlighted by the vertical dashed line

The grid search for the best model parameters was performed for a number *n* of possible features equal to 20, 30, and 40 (nearly 1/3, a half and 2/3 of the original variables), and a number *k* of possible number of clusters equal to 5, 6, 7 and 8. The best option based on silhouette index indicates k=6 and n=20. We then selected the 20 most representative features in the Hospital Dataset, and we grouped them into the 6 clusters indicated by the k-means. The distribution function of the local distances (Fig. 2) being so skewed demonstrates how most of the data points lie close to the center of their cluster, thus sustaining the goodness of the obtained clustering. 10 hospitals resulted as outliers adopting the 95th percentile threshold (dashed line in Fig. 2).

Additionally, to test the robustness of our algorithm, we applied it on the entire dataset skipping the feature selection step, obtaining an average silhouette width of 0.23, and an optimal number of clusters equal to 6. The resulting outliers were 10 hospitals, among which the 6 most distant from their respective centroids belonged to the group identified after feature selection. This coherence testifies for the robustness of our method. Moreover, we include a deeper robustness analysis of our results with different parameters’ settings in the supplementary material (Appendix A.3, Additional file 1).

Three hospitals were selected for an exemplificative application of the second step (H31/public, H51/private, H11/private).

**Fig. 3:**
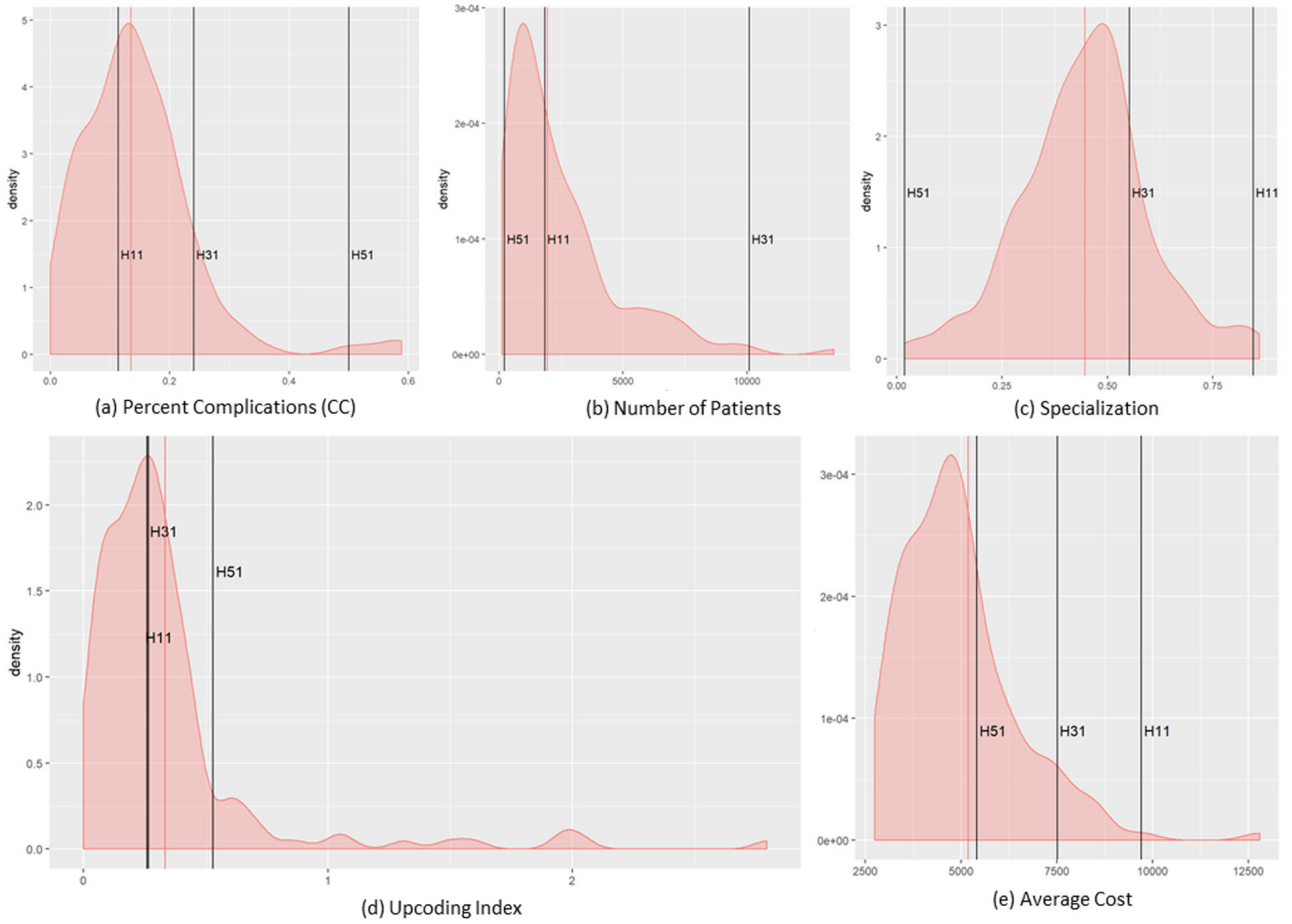
Outliers’ Analysis w.r.t. relevant Features. Distribution of the five Hospital Variables mentioned in “Model Proposal” section for the whole population of hospitals. The vertical lines represent the position of the three outliers (H11, H31, H51) which behavior is analyzed in “Results” section

These three hospitals have been analyzed by evaluating the dimensions defined by the second step of the model. In Fig. 3 the distributions among the whole population of each Hospital variable mentioned in “Model Proposal” section are displayed, and the three hospitals of interest are represented by the vertical lines. Together with the visual representation, in Table 5 we provided the values of the percentile the outliers fall into for each of the analyzed dimensions.

**Table 5:** Percentile of Variables Distributions each analyzed outlier falls into

Variable	H11	H31	H51
Avg Cost	0.994	0.937	0.734
Percent CC	0.399	0.899	0.981
Specialization	0.994	0.816	0
Number of Visits (patients)	0.532	0.994	0.032
Upcoding Index	0.513	0.506	0.890

**Fig. 4:**
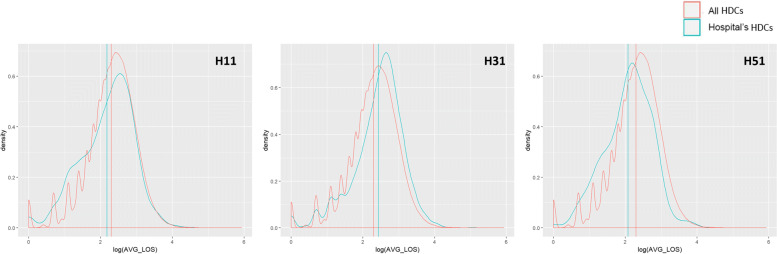
Outliers and Length of Stay. Logarithm of *Length of Stay* distribution for each outlier’s patients compared to all patients’ population

**Fig. 5:**
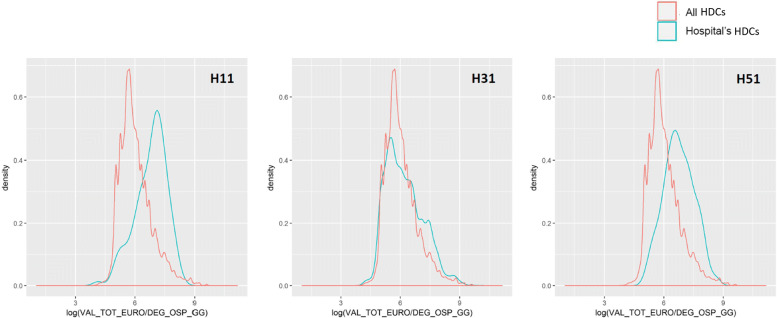
Outliers and Cost/LOS. Logarithm of *Cost/Length of Stay* distribution for each hospitals’ HDCs compared to all HDCs in the dataset

**Fig. 6:**
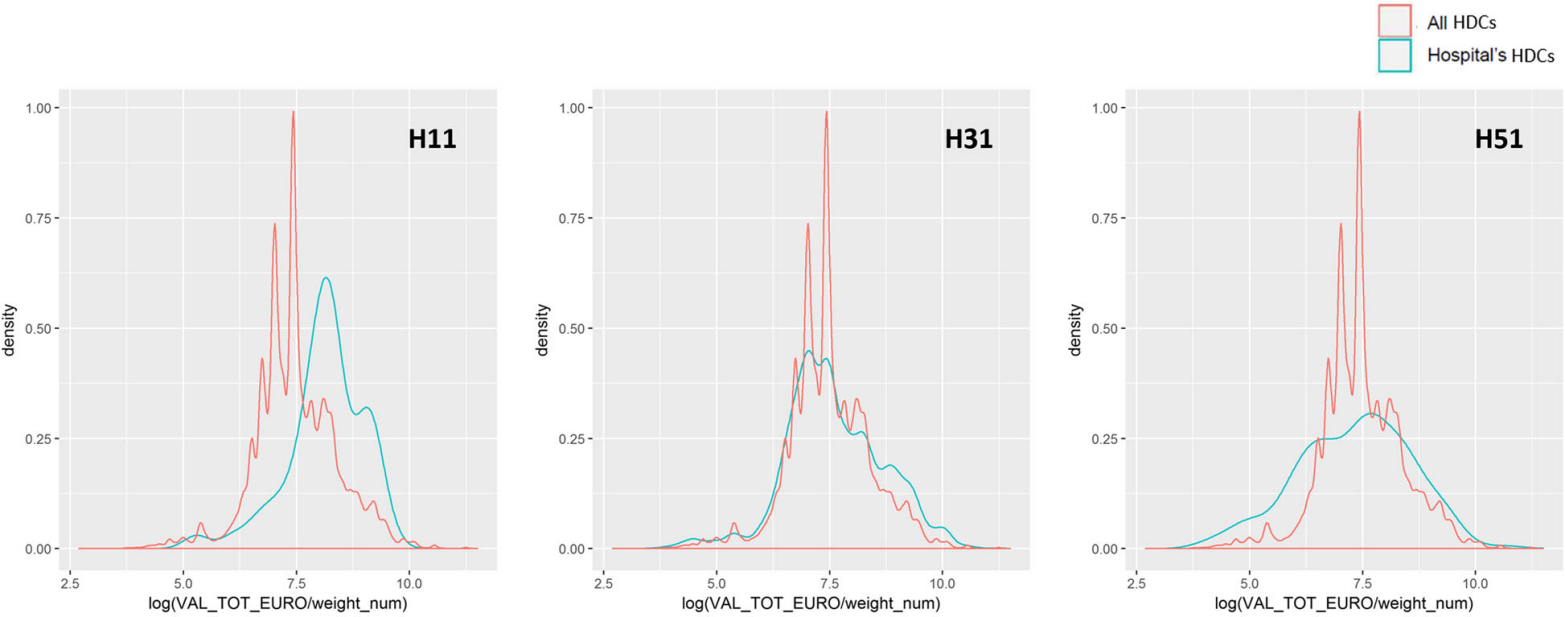
Outliers and Cost/Comorbidity. Logarithm of *Cost/Comorbidity Score* distribution for each hospitals’ HDCs compared to all HDCs in the dataset

As for the cross-validation with the patients’ population, each of the dimensions listed in Table 4 was evaluated, but we decided to report just some exemplificative visualizations. Figure 4 reports the distribution of the average LOS for all patients (in red) - considered as a proxy of complexity of the patient – together with the distribution of LOS for patients treated by the outlying providers (in blue): the more the distribution is right skewed, the more the outlier faced a complex casemix. The vertical lines represent the means of the two distributions. Figures 5 and 6 show the distribution of costs of each registered HDC, w.r.t. the level of complexity of the case: the complexity is represented by the LOS in Fig. 5, and by the comorbidity value in Fig. 6.

**Hospital H31.** This public provider has a lies over the 81^*st*^ percentile of specialization (Table 5), but the mean appears closer to the population’s mean compared to H11. However, this provider receives a much higher number of visits (99th percentile, over ten thousand in three years), suggesting a high perceived value of provided care. Average costs and the percentage of complications are high as well, but the upcoding index falls below the mean (Fig. 3) and almost on the median (Table 5). All together this may suggest that this hospital’s results have been largely driven by its strong specialization and attractiveness. Once performed the cross-validation with the patients’ population, this assumption seems to hold. The patients treated by this provider are on average more complex, according to the LOS distribution in Fig. 4. Moreover, considering the ratios of the reimbursements received for each complexity level (Figs. 5 and 6), this provider does not demonstrate any particular earning above the overall group of providers. For these reasons, this hospital might be eligible for being cleared with no further in-depth investigation.

**Hospital H11 and H51.** These two private providers exhibit much different results compared to the previously mentioned public provider. H51 presents very low levels of specialization (it is the least specialized provider within the population, as shown in Table 5), and it receives a very low number of visits in the considered period. Additionally, both upcoding index and percentage of cases with complications are suspiciously high for a hospital with no clear specialization in the treatment of HF. H11 on the contrary seems to be much more specialized (99th percentile) and presents a low percentage of complications together with an average upcoding index. However, when cross-validated with their patients’ population, both private providers suggest some suspect behavior. They both show lower levels of LOS compared to the population (Fig. 4), but their reimbursements associated with the complexity of their patients appear higher than the average (w.r.t. both LOS and comorbidity for H11, and w.r.t. LOS only for H51 - Figs. 5 and 6). Even though the two cases are different and may be grounded on different causes, both providers seem to be worthy of a deeper investigation, by someone who may add its domain specific expertise to decide whether an actual fraudulent behavior is in place.

The three cases were selected for illustrative purposes. It is important to underline that none of the conclusion drawn from this last passage of the model are in any way a definitive judgment of the existence or not of a fraudulent behavior. The aim of this last step is to provide auditors with a useful set of information to ease their appraisal process, and reduce the risk of wasting their effort on false positive cases.

## Discussion

The objective of this study was proposing a novel methodology to support the preliminary screening phases performed by auditors in the fight against upcoding fraud. Our aim was that of building an easy-to-use and robust tool. The procedure is indeed automated for most part (Step 1 entails a semi-automated algorithm to create a list of suspects), requiring very little statistical knowledge and manipulation on the user’s side. We chose to exploit a simple clustering algorithm (i.e. k-means) to identify providers that lie distant from their peers, by automatically searching for K (with K optimized via grid search) *locally consistent* groups of neighboring hospitals that supposedly behave similarly, so that those lying more distant from such reference groups can be flagged as suspects.

There are several clustering algorithms that may be considered as alternatives to the k-means. Among others, several different hierarchical clustering methods (both agglomerative and divisive) [28, 30], together with more recent efficient and effective approaches such as DBSCAN [31], spectral methods [32], or the modern Deep Learning-based approaches [33]. All these algorithms present peculiarities that allow them to separate at best the clusters intrinsically available in data, up to a different extent of complexity in the shape of the aforementioned clusters. However, at the same time, most of them require some specific assumptions on cluster shapes or need specific interventions from the user side. For instance, DBSCAN is quite efficient as a method and has the pro of not requiring the number of clusters to be defined in advance, but it fails to identify clusters if density varies; another example could be SLINK [30], an optimally efficient single-linkage clustering, that tends to produce long thin clusters in which nearby elements of the same cluster have small distances, but elements at opposite ends of a cluster may be much farther from each other than two elements of other clusters; regarding tailored interventions we might mention the dendrogram analysis for agglomerative/divisive clustering to define clusters, or the hyperparameter choice and training tailoring for deep learning-based approaches.

However, as we explained in the “Methods” section (under *Model Proposal*), our proposition lies in a completely unsupervised setting, where (i) no prior assumption can be made, (ii) we are not interested in classifying providers in any specific group, and (iii) our objective is to keep complexity on the user-side to the minimum, while (iv) efficiently providing a scalable and reliable algorithm that searches for groups that need to be consistent only on a *local* basis. All this considered, the simple k-means algorithm results the faster in terms of computational time, the most robust to varying densities of clusters and high dimensionality of data, and the simplest to be included within a wider algorithm that automatically selects the best features subsets and the best number of clusters (as in the grid search passage described in the following), to define groups of neighboring providers that are supposedly similar on most dimensions of the multidimensional space they lie into.

The grid search step before the application of the clustering technique makes the algorithm flexible to the different settings (where sample size might be smaller, comparable or bigger than dimensionality), identifying programmatically the model obtaining the best clustering of observations. Since the computational intensity of the search grows together with the number of hospitals and the number of possible combinations to be evaluated, the user is suggested to choose a subset of parameters (i.e. the dimension of the grid). However, it has to be noted that the number of variables collected in the Hospital Dataset is bounded by the limited number of DRGs within each disease category, while the number of hospitals analyzed might grow indefinitely. This reduces the need for a feature selection passage, in case its application would be too computationally expensive, or the user would not feel confident in listing the parameters to be evaluated. We empirically demonstrated that even skipping the feature selection our proposed algorithm remains a valid support. For what concerns Step 2, the provided results should be considered a demo application, deep diving into the analysis of three exemplificative outliers, taking the role of the auditors and showcasing the process they would follow. Moreover, the provided results seem reasonable, supporting the usefulness of the overall process in supporting the auditing process, and of the dashboards in supporting the auditors’ decisions. Indeed, the methodology here proposed was tested on real data concerning a complex chronic disease like Heart Failure, to verify whether significant insights might be inferred by the obtained results. The analysis on the three outlying healthcare providers provided clear suggestions to a potential third party user concerning whether the suspects should be studied deeper in order to unveil an actual fraud. The main novelty of our approach regards the definition of several fraud related indexes and an array of variables that represent the coding behavior of providers, together with a simple yet effective method to identify suspicious behaviors that do not stand out by exceeding on any specific dimension. It is indeed the ensemble of searching for cluster-specific outliers (where *clusters* have to be intended as *locally consistent* and *locally similar* groups of providers) and using these derived variables to spot them, the real novelty and value introduced by our methodology. Moreover, an additional aspect of novelty w.r.t. other *behavioral* methods, such as the work in [17], is the inclusion of a follow-up step (Step 2) that is mostly human-decision based, and directly controls for the risk of false positives derived by the search for less evident fraudsters.

Although the proposed algorithm is promising, there a few limitations that open avenues for further research. First, our raw data cover a time span of three years. Although this time span is enough for the proof of concept of our methodology, further studies should gather raw data about a longer period in order to verify the capability of better profiling patients and hospital misbehaviours. Second, our results have not been validated by domain experts, being the validation step out of our research protocol. This situation is frequent when raw data are owned by public organizations and/or private companies that want to keep full control of the actual identification of fraudulent behaviours, being this information very sensitive. Thus researchers are often allowed to access to unlabelled data and obliged to develop unsupervised models, limiting their studies to proof of concepts. Further studies should try to go beyond this limitation by including the validation step within the research protocol and by persuading the owners of raw data of the many advantages of including researchers also in the validation step. Third, our study relies on hospital discharge charts. Although these administrative data are rich enough to provide auditors with suggestions about potential upcoding frauds, further studies should consider to include other knowledge sources – e.g., Electronic Medical Records (EMRs) – that offer more clinical data that might help better profiling patients in terms of their health status and of what care are they receiving.

## Conclusions

Despite the limitations, our proposed methodology contributes to the still limited literature about behavioral modelling for fraud detection, with an attention to *cautious* fraudsters lying in between more evident cases. In fact, our algorithm is novel (as described in the “Discussion” section), scalable and interpretable. The clear interpretability (and understandability) of both the algorithm and its results, supported by visualizations of easily recognizable features, makes up for the potential lack of precision (and proper evaluation metrics, such as “accuracy”, “F1-score” etc.) that only a supervised classifier could obtain. Indeed, we are providing a powerful and flexible data-miner, whose aim is to make educated guesses (smarter with respect to the actual random selection of providers to be audited, as it currently happens where our Case Study data originates) on cases to be further and deeply investigated. We leave the judgement completely in the hand of auditors and users, basically providing them with better-shaped information to support their decision, on a highly interesting subset of a huge amount of data. Despite the proof of concept of our method was developed in the Italian context, studying the behavior of hospitals adopting regional administrative data, it is generally adaptive to any kind of system and any disease of interest. Additionally, the model is based on an existing and widely adopted coding mechanism (DRG): as such, it can be easily applied to a large number of databases. Moreover, our methodology is easy-to-use as it does not need extensive interventions or statistical knowledge on the user’s side. Finally, the proposed algorithm can help auditors by reducing (i) the amount of time needed for the initial screening and (ii) the unwanted variation of results due to skills of different auditors (as for any operator-dependent tasks).

## Supplementary information

**Additional file 1** It is divided into two sections:
Section A.1 Contains details about the Patients Dataset
Section A.2 Contains details about the Hospital Dataset, the original features it contained and how additional indexes were computed.
Section A.3 Contains the experiments (and relative results) carried out to verify the robustness of Step 1 of the proposed methodology in searching for outliers, comparing results for different features’ subset dimensions and different numbers of clusters.

## Data Availability

The data that support the findings of this study are available from Regione Lombardia but restrictions apply to the availability of these data, which were used under license for the current study, and so are not publicly available. Data are however available from the authors upon reasonable request and with permission of Regione Lombardia.

## Abbreviations

DRG: Diagnosis related group
HDC: Hospital discharge charts
ICD: International classification of diseases
IPPS: Inpatient prospective payment system
HF: Heart failure
CI: Comorbidity index
LOS: Length of stay
HAC: Hierarchical agglomerative clustering
PFA: Principal feature analysis
EMR: Electronic medical records

## Footnotes

1 A program based on ICD (International Classification of Diseases) diagnoses, procedures, age, sex, discharge status, and the presence of complications or comorbidities. The DRG-Grouper is used to calculate payments to cover operating costs for inpatient hospital stays. Under the inpatient prospective payment system (IPPS) payment weights are assigned to each DRG based on average resources used to treat patients in that DRG.

## References

[1] OECD. Tackling Wasteful Spending on Health; 2017, p. 304. https://doi.org/10.1787/9789264266414-en. https://www.oecd-ilibrary.org/content/publication/9789264266414-en.

[2] Steinbusch PJ, Oostenbrink JB, Zuurbier JJ, Schaepkens FJ (2007). The risk of upcoding in casemix systems: a comparative study. Health Policy.

[3] Luo W, Gallagher M. Unsupervised drg upcoding detection in healthcare databases. In: 2010 IEEE International Conference on Data Mining Workshops. IEEE: 2010. p. 600–5. 10.1109/icdmw.2010.108.

[4] Berta P, Callea G, Martini G, Vittadini G (2010). The effects of upcoding, cream skimming and readmissions on the italian hospitals efficiency: A population-based investigation. Econ Model.

[5] Bauder R, Khoshgoftaar TM, Seliya N (2017). A survey on the state of healthcare upcoding fraud analysis and detection. Health Serv Outcomes Res Methodol.

[6] Simborg DW. DRG creep: a new hospital-acquired disease. Mass Med Soc. 1981. 10.1056/nejm198106253042611. 10.1056/NEJM198106253042611 7015136

[7] Silverman E, Skinner J (2004). Medicare upcoding and hospital ownership. J Health Econ.

[8] O’malley KJ, Cook KF, Price MD, Wildes KR, Hurdle JF, Ashton CM (2005). Measuring diagnoses: Icd code accuracy. Health Serv Res.

[9] Pearson RA, Murray W, Mettenmeyer T (2006). Finding anomalies in medicare. Electron J Health Inf.

[10] Kumar M, Ghani R, Mei Z-S. Data mining to predict and prevent errors in health insurance claims processing. In: Proceedings of the 16th ACM SIGKDD International Conference on Knowledge Discovery and Data Mining: 2010. p. 65–74. 10.1145/1835804.1835816.

[11] Francis C, Pepper N, Strong H. Using support vector machines to detect medical fraud and abuse. In: 2011 Annual International Conference of the IEEE Engineering in Medicine and Biology Society. IEEE: 2011. p. 8291–4. 10.1109/iembs.2011.6092044. 10.1109/IEMBS.2011.6092044 22256268

[12] Kirlidog M, Asuk C (2012). A fraud detection approach with data mining in health insurance. Proc-Soc Behav Sci.

[13] Joudaki H, Rashidian A, Minaei-Bidgoli B, Mahmoodi M, Geraili B, Nasiri M, Arab M (2015). Using data mining to detect health care fraud and abuse: a review of literature. Global J Health Sci.

[14] Yang W-S, Hwang S-Y (2006). A process-mining framework for the detection of healthcare fraud and abuse. Expert Syst Appl.

[15] Shan Y, Jeacocke D, Murray DW, Sutinen A. Mining medical specialist billing patterns for health service management. In: Proceedings of the 7th Australasian Data Mining Conference-Volume 87. AUS: Australian Computer Society, Inc.: 2008. p. 105–10.

[16] Shan Y, Murray DW, Sutinen A. Discovering inappropriate billings with local density based outlier detection method. In: Proceedings of the Eighth Australasian Data Mining Conference-Volume 101. Australian Computer Society, Inc.; AUS: 2009. p. 93–8.

[17] Musal RM (2010). Two models to investigate medicare fraud within unsupervised databases. Expert Syst Appl.

[18] Tang M, Mendis BSU, Murray DW, Hu Y, Sutinen A. Unsupervised fraud detection in medicare australia. In: Proceedings of the Ninth Australasian Data Mining Conference-Volume 121. AUS: Australian Computer Society, Inc.: 2011. p. 103–10.

[19] Shin H, Park H, Lee J, Jhee WC (2012). A scoring model to detect abusive billing patterns in health insurance claims. Exp Syst Appl.

[20] Liu Q, Vasarhelyi M. Healthcare fraud detection: A survey and a clustering model incorporating geo-location information. In: 29th World Continuous Auditing and Reporting Symposium (29WCARS), Brisbane, Australia: 2013.

[21] Konijn RM, Duivesteijn W, Kowalczyk W, Knobbe A. Discovering local subgroups, with an application to fraud detection. In: Pacific-Asia Conference on Knowledge Discovery and Data Mining. Springer: 2013. p. 1–12. 10.1007/978-3-642-37453-1_1.

[22] Konijn RM, Duivesteijn W, Meeng M, Knobbe A (2015). Cost-based quality measures in subgroup discovery. J Intell Inf Syst.

[23] van Capelleveen G, Poel M, Mueller RM, Thornton D, van Hillegersberg J (2016). Outlier detection in healthcare fraud: A case study in the medicaid dental domain. Int J Account Inf Syst.

[24] Bolton RJ, Hand DJ (2002). Statistical fraud detection: A review. Stat Sci.

[25] Gagne JJ, Glynn RJ, Avorn J, Levin R, Schneeweiss S (2011). A combined comorbidity score predicted mortality in elderly patients better than existing scores. J Clin Epidemiol.

[26] Cheng TC, Haisken-DeNew JP, Yong J (2015). Cream skimming and hospital transfers in a mixed public-private system. Soc Sci Med.

[27] Ekin T, Ieva F, Ruggeri F, Soyer R (2017). On the use of the concentration function in medical fraud assessment. Am Stat.

[28] Rokach L, Maimon O. Clustering methods. In: Data Mining and Knowledge Discovery Handbook. Springer: 2005. p. 321–52. 10.1007/b107408.

[29] Lu Y, Cohen I, Zhou XS, Tian Q. Feature selection using principal feature analysis. In: Proceedings of the 15th ACM International Conference on Multimedia: 2007. p. 301–304. 10.1145/1291233.1291297.

[30] Sibson R (1973). Slink: an optimally efficient algorithm for the single-link cluster method. Comput J.

[31] Ester M, Kriegel H-P, Sander J, Xu X (1996). A density-based algorithm for discovering clusters in large spatial databases with noise. Kdd.

[32] Filippone M, Camastra F, Masulli F, Rovetta S (2008). A survey of kernel and spectral methods for clustering. Pattern Recog.

[33] Karim MR, Beyan O, Zappa A, Costa IG, Rebholz-Schuhmann D, Cochez M, Decker S. Deep learning-based clustering approaches for bioinformatics. Brief Bioinforma. 2020. https://doi.org/10.1093/bib/bbz170, bbz170. https://academic.oup.com/bib/advance-article-pdf/doi/10.1093/bib/bbz170/32313172/bbz170.pdf. 10.1093/bib/bbz170 PMC7820885 32008043

