# Supplementary material for: Data mining application to healthcare fraud detection: a two-step unsupervised clustering method for outlier detection with administrative databases
Source: BMC Med Inform Decis Mak. 2020 Jul 14;20:160. doi: 10.1186/s12911-020-01143-9 (PMC7362640; doi:10.1186/s12911-020-01143-9)
Supplement: Supplementary file 1 — Additional file 1 It is divided into two sections: Section A.1 Contains details about the Patients Dataset Section A.2 Contains details about the Hospital Dataset, the original features it contained and how additional indexes were computed. Section A.3 Contains the experiments (and relative results) carried out to verify the robustness of Step 1 of the proposed methodology in searching for outliers, comparing results for different features’ subset dimensions and different numbers of clusters. [file 12911_2020_1143_MOESM1_ESM.pdf]

Data Mining Application to Healthcare Fraud Detection: A Two-Step Unsupervised Clustering Method for Outlier Detection with Administrative Databases

Full list of author information is available at the end of the article

Appendix

A.1 Patients Dataset Description

In Table T.A.1 are listed all the variables available within the Patients Dataset. For those variables where more than a value was available due to repeated nature of the data (e.g. we have an 'age' value for each record related to a patient, being a record a single hospitalization) we decided to resume the information into a single value (e.g. among all the age values, we reported the age at first hospitalization. For costs and LOS we took the averages, etc.).

VARIABLE	DESCRIPTION
COD_FISC	Unique identification number (ID), which has been masked for patients' privacy reasons
BIRTH_DATE	Date of birth
DEATH_DATE	Date of death, available for those patient deceased during the time frame considered
AGE	Age at the time of first available HDC for each patient
SEX	Gender of the patient
COMUNE_RESID	Municipality of residency of the patient
TOT_HOSPITALIZATIONS	Total number of hospitalizations in the considered period
AVG_COST	Average cost of each hospitalization registered for the patient. Calculated as the sum of reimbursement received for each hospitalization (as reported on the HDC) divided by the number of HDCs
AVG_LOS	Average lenght of each patient's hospitalizations, in days
TOT_COST	Sum of all reimbursements received for each hospitalization
TOT_COMORB	Sum of all Comorbidity Indexes for each hospitalization of the patient
N_HOSPITALS	Number of different hospitals where the patient was hospitalized

Table T.A.1 Patient Dataset details

A.2 **Hospital Dataset Description**

For each hospital, before adding the fraud related variables suggested by the literature, we had the following information:

- ID;
- Number of Patients: counted as the number of HF patients treated, to be considered as proxy of hospital's dimension and specialization in the treatment of HF disease;
- Total Cost: sum of all reimbursements received in the considered time frame;
- Total Comorbidity Index: sum of all comorbidity scores for all HDCs registered by the provider;
- Average Cost: The average reimbursement received per hospitalization;
- Average Comordibity Score: the average comordibity per hospitalization;
- Flag Public/Private: a flag indicating whether the hospital is a public or private provider.

In Table T.A.2 we report some summary statistics about the aforementioned variables (mean and standard deviation for numeric variables, count and percentage of the total for categorical variables.)

Variable	Descriptive Statistics	
	Mean	Std Dev
**Number of Patients**	2,149	2,244.05
**Total Cost**	11,306,190 [€]	13,620,588 [€]
**Total Comorbidity Index**	4,053.8	4,459.26
**Average Cost**	5,009 [€]	1,643.4 [€]
**Average Comorbidity Score**	1.7218	0.528
	Count	Percentage
**Flag Public**	104	57 %
**Flag Private**	79	43 %

Table T.A.2 Summary statistics of original hospitals' data

To these variables we added a set of features inspired by our literature review about fraud detection in the healthcare domain. In the following we'll describe how each of those variables was estimated and added to the Hospital Dataset.

Silverman's Upcoding Index. The first estimated index is the *Upcoding Index* as expressed in [1]: the ratio between the number of most remunerative DRGs coded in treatment of a disease and the sum of all DRGs related to such disease. In order to repurpose this index in our context we traced the Pareto Curve of reimbursement tariffs associated to each DRG[1], and we defined as 'heavy DRGs' those accounting for 60% of the value of all HF DRGs together (7 were selected, out of 44). The rate of incidence of those DRGs ($heavyDRG_i$, $i = 1, ..., I$) on the overall registrations for HF cases (DRG_k, $k = 1, ..., K$ being the total number of HF-DRGs) was computed for each hospital ($h = 1,...,H$) as:

[1]The tariffs' list made public by the Italian Healthcare Ministry was used as reference for both this and the next estimated indexes. It contains for each DRG code its description and the reimbursement due to the hospital.

$$UpcodingIndex_h = \frac{\sum_i heavyDRG_{hi}}{\sum_k DRG_{kh}} \tag{E.A.1}$$

Berta's Upcoding Index. The second indicator is an Upcoding Index proposed by Berta *et al.* [2]. It improves the previous one with the additional 'Comorbidity' load, which adjusts results taking into consideration patients' illness status. Differently from the original version proposed in [2], we did not consider the time trend of the indicator, but we collapsed the information over the years. For each hospital ($h = 1, ..., H$):

$$UPCODING_h = \frac{S_h^C}{S^C} * \frac{1}{CI_h} \tag{E.A.2}$$

Being S_i^C the share of discharges with complications over the total number of discharges in hospital h ($\sum_j HDC_{jh}^C / \sum_i HDC_{hi}$, where $j = 1, ..., J$ are the discharges registered with complications, while $i = 1, ..., I$ are all discharges). This share is compared to the share computed at regional level:

$$S^C = \frac{\sum_h \sum_j HDC_{jh}^C}{\sum_h \sum_i HDC_{hi}} \tag{E.A.3}$$

The ratio shows whether hospital h is treating more complex cases than regional average [2]. The ratio is then divided by the Comorbidity Index (CI_h) of the hospital, estimated as the average CI of the treated patients. To compute the total discharges
($\sum_h \sum_i HDC_{hi}$) we considered all HF-DRGs, while the cases with complications (HDC_{hi}^C) were selected from the regional tariff's list among those reporting '*complications*' or
'*Acute Myocardial Infarction (AMI)*' within the description. AMI is an exceptionally critical condition that alone drives the cost of the treatment higher, even if no complications are specified within the description. For that reason, we decided to include such DRGs in the group of those 'with complications'. The total number of DRGs with complications is reported in the ***DRG_CC***, while S^C value is stored in the ***percent_CC*** variable.

Behavioral Indexes (r_{hi}). A particular attention was then devoted to Ekin *et al.* [3], since their contribution suggested a way to represent providers' behavior, instead of estimating a single measure of fraudulence.
In order to model hospitals' behavior, we estimated the values of r_{hi} similarly to how it was proposed in [3], i.e., as the ratio between the probability that the hospital h bills the treatment i, and the probability of the whole population to bill for the treatment i. We decided to exploit the concept to represent how each hospital

behaved in the treatment of a particular disease through a set of indexes. Since DRGs are calculated by the grouper on the basis of what kind of treatments were performed and the status of the patient, they can be considered a good proxy of each case faced by the provider, and how the provider behaved in the treatment of the patient. For this reason, we decided to adopt DRG codes in our estimation of r_{hi}.
Therefore, for each HF-related DRG ($i = 1, ..., I$) in each hospital ($h = 1, ..., H$), the r_{hi} value was estimated as

$$r_{hi} = \frac{\frac{\sum_k DRG_{ikh}}{\sum_i \sum_k DRG_{ikh}}}{\frac{\sum_h \sum_k DRG_{ikh}}{\sum_h \sum_i \sum_k DRG_{ikh}}} \tag{E.A.4}$$

Where $k = 1, ..., K$ are the records where DRG_i is registered. The string of r_{hi} values for each hospital represents how the hospital behaves in terms of coding treatments for HF patients.

Readmission Index. This indicator (***Readm_Indx***) estimates whether the hospital perpetrated an opportunistic behavior by discharging patients and readmitting them after a short period of time in order to maximize the reimbursements received. In [2] the authors define the index as the ratio between the number of readmission in the same hospital for the same MDC (HF in our case) within Δ days from discharge, and the total number of admissions to the hospital h.
In our application of their index first of all the idle time between each discharge and subsequent readmission in the same hospital was computed, for HF-related causes. Then, a time frame of 15 days was chosen as Δ.
Similarly, the Number of Suspect Readmissions index (***n_Susp_Readm***) is the count of readmissions in a timeframe of 20 days.

Same Day Separation. Number of HF-related hospitalizations where the patient was discharged the same day it was hospitalized (***same_day_sep***). Together with the Readmission Index this measure might suggest an opportunistic behavior, or a low quality of provided care.

In Table T.A.3 are listed all the variables within the Hospital Dataset after the estimation of the additional fraud-related indexes suggested by literature. The table reports the complete list of features which were fed into the k-means algorithm, together with a brief explanation of their meaning.
The total number of features in the Hospital Dataset is 64, with 47 variables dedicated to *behavioral* indices (r_i, stored in **DRG_[n]_r**, with $n = 1, ..., 47$).

Hospitals' Variables for Clustering	
**avg_comorbidity**	Average comorbidity index
**avg_cost**	Average value for HDC
**n_patients**	Number of cases treated
**Tot_cost**	Sum of all costs of HDCs
**tot_comorbidity**	Sum of all comorbidity indexes
**heavy_HF_DRG_n**	Number of heavy DRGs HF-related
**HF_DRG_n**	Number of HF-related HDCs
**Silv_Up_index**	Upcoding index according to Silverman, computed as in (E.A.1)
**percent_CC**	Percentage of DRGs with complications as in (E.A.3)
**DRG_CC**	Number of DRGs with complications
**Berta_up_index**	Upcoding index according to Berta, computed as in (E.A.2)
**n_susp_readm**	Number of readmission cases in the same hospital within 20 days
**Readm_Indx**	Percentage of suspect readmission cases with $\Delta = 15days$
**same_day_sep**	Number of cases in which the patient was dismissed the same day it was hospitalized
**avg_r**	Average of r values
**TOTcost_on_comorb**	Total costs over total comorbidities
**avg_age**	Average age of patients
**DRG_[n]_r**	All r values for each HF-related [n] DRG, computed as in (E.A.4)

Table T.A.3 List of variables within Hospital Dataset

A.3 **Robustness Analysis in the search for outliers**

In the paper it is mentioned how the outliers identified using all features overlap with those identified after features selection (Section Results). However, the discussion on the robustness of the methodology could be further expanded.
To strengthen our position, we analysed all the lists of outliers identified with the parameters n (number of features) and k (number of clusters) used in the grid search for feature selection (Table T.A.4).

First of all, note that by applying different parameters, the number of identified outliers remains stable. The reader should keep in mind that these outliers were selected by imposing a 95th percentile threshold on the distribution of within-cluster distances. The fact that the method robustly identifies 10 outliers despite the parameters' configuration is likely due to the fact that the configurations of the various clusters is not changing significantly, and the distribution keeps having a similar shape to the one shown in Figure 2 in the paper.

We then verified how many different outliers were highlighted with the various combinations of parameters, and we recognized only 18 different IDs, among 12 lists of 10 outliers. This small subset of providers deemed suspicious by our method testing several configurations, among the 183 available in the dataset, testifies once again in favour of the robustness of the proposed methodology.

To deepen the analysis, we counted in how many outliers' lists compared each one of the IDs. Results are reported in Figure F.A.1. Note that more than a half of the IDs appear within more than 50% of these lists, with some specific provider (such as number 51, 54 or 178) consistently recognized as outlier despite the different values of k and n.
As mentioned, the plot in Figure F.A.1 was built by considering all different configurations. However, one may argue that the consistency of results should be demonstrated comparing lists built under the same number of clusters, as the concept of 'outlier distant from its peers' best applies in this case.
For this reason, and to test the robustness of the feature selection passage as well, we

Number of Variables	K	Outliers ID									
20	5	21	51	52	54	97	131	140	159	167	176
20	6	21	51	54	88	131	151	157	159	176	178
20	7	21	51	52	54	97	151	157	159	167	178
20	8	21	52	97	140	141	151	157	159	176	178
30	5	21	51	52	54	97	131	165	167	176	178
30	6	21	51	54	97	131	140	141	165	167	178
30	7	21	43	51	52	54	97	131	165	167	178
30	8	21	43	52	54	97	131	140	165	167	178
40	5	51	52	54	97	98	131	165	167	176	178
40	6	51	52	98	131	140	141	165	167	176	178
40	7	51	52	54	97	98	131	165	167	176	178
40	8	51	52	54	97	98	131	140	165	167	178

Table T.A.4 Outliers lists identified by using different number of variables (n) or different number of clusters (k)

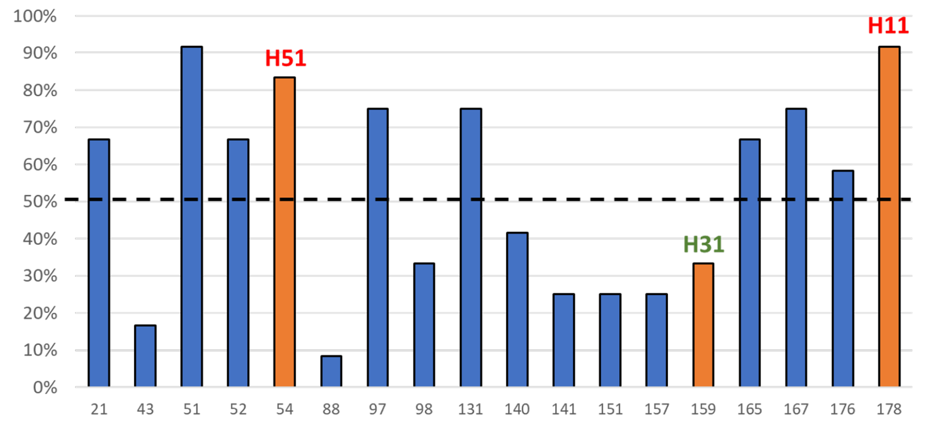

Figure F.A.1 Percentage of appearence of each Provider ID within the outliers' lists identified with the different combinations of k and n described in Table T.A.4.

created Table T.A.5 for reference, where the same lists of Table T.A.4 are grouped by the K value. In Figure F.A.2 the same plot as in Figure F.A.1 restricted to the outliers' lists identified for $K = 6$ (as this is the parameter setting described in the paper).

Note that the plots are quite similar, with some IDs appearing 100% of the times in the lists created with $n = [20, 30, 40]$.

One last consideration might be pointed out regarding the three deepened outliers in the Results Section describing Step 2. Indeed, it is interesting to highlight how the two providers that were still suspicious after Step 2 are those belonging to the majority of lists. This would mean that by slightly changing the parameters configuration, we would have probably still ended up suggesting to a potential auditor the two most suspect providers, excluding only the one that was justified in its outlierness after all.

Finally, a last remark has to be done. Unfortunately, due to the restrictions the project is constrained, the aim of the analysis requested to the group was the development of a proof of concept for a large-scale application to administrative data. Therefore, since we have not been provided with the actual labels of data, it is not

Number of Variables	K	Outliers ID									
20	5	21	51	52	54	97	131	140	159	167	176
30	5	21	51	52	54	97	131	165	167	176	178
40	5	51	52	54	97	98	131	165	167	176	178
20	6	21	51	54	88	131	151	157	159	176	178
30	6	21	51	54	97	131	140	141	165	167	178
40	6	51	52	98	131	140	141	165	167	176	178
20	7	21	51	52	54	97	151	157	159	167	178
30	7	21	43	51	52	54	97	131	165	167	178
40	7	51	52	54	97	98	131	165	167	176	178
20	8	21	52	97	140	141	151	157	159	176	178
30	8	21	43	52	54	97	131	140	165	167	178
40	8	51	52	54	97	98	131	140	165	167	178

Table T.A.5 Outliers' Lists reported in Table T.A.4, reordered and grouped on the basis of the number of clusters (K) imposed to the k-means algorithm.

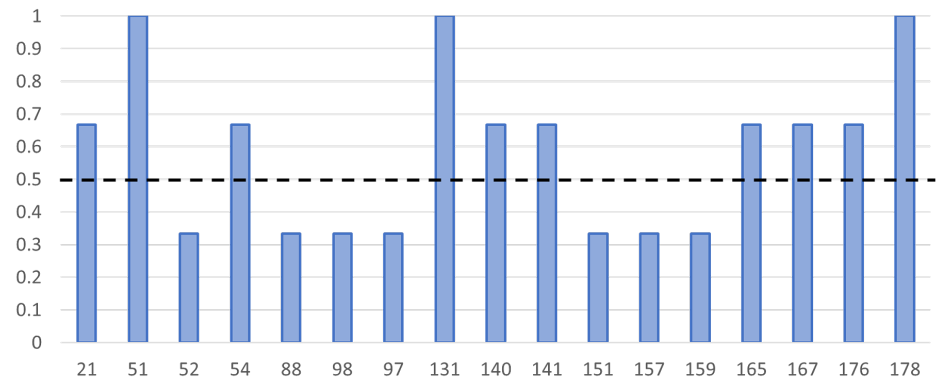

Figure F.A.2 Percentage of appearence of each Provider ID within the outliers' lists identified with k = 6 and varying values of n (selected features' subset dimension), referred to the grouping described in Table T.A.5.

possible to compute common indexes of robustness or accuracy. For this reason, we are definitely not making any final judgement on any of the discussed outliers, we just suggest the way the algorithm should be used to this aim. However, all the empirical evidence provided, in these analyses and in the paper, seem to suggest that our methodology is robust and could effectively support the surveillance tasks of a human auditor.

Author details

References

1. Silverman, E., Skinner, J.: Medicare upcoding and hospital ownership. Journal of health economics **23**(2), 369–389 (2004)
2. Berta, P., Callea, G., Martini, G., Vittadini, G.: The effects of upcoding, cream skimming and readmissions on the italian hospitals efficiency: A population-based investigation. Economic Modelling **27**(4), 812–821 (2010)
3. Ekin, T., Ieva, F., Ruggeri, F., Soyer, R.: On the use of the concentration function in medical fraud assessment. The American Statistician **71**(3), 236–241 (2017)
